# Supplementary material for: Conditional chemoconnectomics (cCCTomics) as a strategy for efficient and conditional targeting of chemical transmission
Source: eLife. 2024 Apr 30;12:RP91927. doi: 10.7554/eLife.91927 (PMC11060718; doi:10.7554/eLife.91927)
Supplement: Figure 3—source data 3. [file elife-91927-fig3-data3.zip › Figure 3 source data3 part 1.zip!/Figure 3-Source data 3-part1/1071f3-1-CRISPResso_Report_jvi77nmx.zip!/CRISPResso_on_1071f3_2/2b.Nucleotide_percentage_quilt_around_sgRNA_actactacatctccgtggag.pdf]

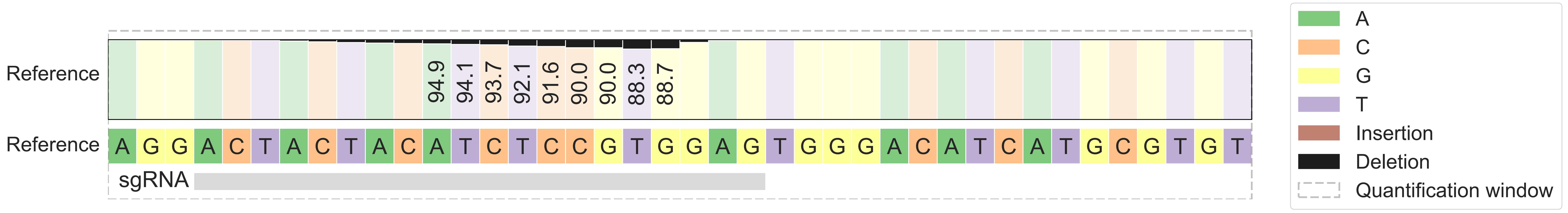
Reference
94.9
94.1
93.7
92.1
91.6
90.0
90.0
88.3
88.7
Reference
A G G A C T A C T A C A T C T C C G T G G A G T G G G A C A T C A T G C G T G T
sgRNA
A
C
G
T
Insertion
Deletion
Quantification window
